# hTR repressor-related gene on human chromosome 10p15.1

**DOI:** 10.1054/bjoc.2001.2121

**Published:** 2001-11

**Authors:** N Miura, N Onuki, A Rathi, A Virmani, S Nakamoto, Y Kishimoto, Y Murawaki, H Kawasaki, J Hasegawa, M Oshimura, W D Travis, A F Gazdar

**Affiliations:** Hamon Center for Therapeutic Oncology Research, Department of Pathology, University of Texas Southwestern Medical Center, Dallas, Texas, 75390-8593, USA; Department of Surgery, Tokyo Women's Medical College, Tokyo, 162-8666, Japan; Department of Laboratory Medicine, Tottori Prefectural Central Hospital, Tottori, Tottori, 680-0901, Japan; Department of Clinical Pharmacology, Tottori University, Yonago, Tottori, 683-8503, Japan; The Second Department of Internal Medicine, Tottori University, Yonago, Tottori, 683-8503, Japan; Department of Molecular and Cell Genetics, School of Life of Sciences, Faculty of Medicine, Tottori University, Yonago, Tottori, 683-8503, Japan; Department of Pulmonary and Mediastinal Pathology, Armed Forces Institute of Pathology, Washington, DC, 20306-6000, USA

**Keywords:** hTR, in situ hybridization, mapping, chromosome 10p15, telomerase repressor gene, loss of heterozygosity

## Abstract

Somatic cells express genes that suppress telomerase activity and these genes may be inactivated in tumour cells. We postulated that cancer cells acquire immortality by activation of telomerase by the loss of such a gene. We have reported recently that a telomerase repressor gene may be located on 10p15.1 by deletion mapping using microcell-mediated chromosome transfer (MMCT), radiated microcell fusion (RMF), fluorescent in situ hybridization (FISH) and STS analysis. To independently confirm this result, we correlated expression of RNA component of telomerase (hTR) as a marker of telomerase expression by in situ hybridization with allelic loss in pulmonary carcinoid tumours. Unlike most malignant tumours, pulmonary carcinoids (which are low-grade malignant tumours) are heterogeneous for telomerase expression. Loss of 5 closely spaced polymorphic markers on 10p15.1, especially D10S1728, were highly correlated with hTR expression. In an additional experiment, 10p15.1 showed higher and more significant correlation than any region of 3p where it has been predicted as another chromosomal location of telomerase repressor with allelic loss of the region. Our findings strongly suggest that 10p15.1 harbours a gene involved in repression of telomerase RNA component in human somatic cells and each putative repressor (on 3p and 10p) may act independently. © 2001 Cancer Research Campaign   http://www.bjcancer.com


*British Journal of Cancer* (2001) **85**(10), 1510–1514
© 2001 Cancer Research Campaign
doi: 10.1054/ bjoc.2001.2121, available online at http://www.idealibrary.com on IDEAL

### hTR repressor-related gene on human chromosome 10p15.1

**N Miura[1], N Onuki[2], A Rathi[1], A Virmani[1], S Nakamoto[3], Y Kishimoto[4], Y Murawaki[5], H Kawasaki[5], J Hasegawa[4], M Oshimura[6], WD Travis[7] and AF Gazdar[1]**

[1]Hamon Center for Therapeutic Oncology Research, Department of Pathology, University of Texas Southwestern Medical Center, Dallas, Texas 75390-8593, USA; [2]Department of Surgery, Tokyo Women's Medical College, Tokyo 162-8666, Japan; [3]Department of Laboratory Medicine, Tottori Prefectural Central Hospital, Tottori, Tottori 680-0901, Japan; [4]Department of Clinical Pharmacology, Tottori University, Yonago, Tottori 683-8503, Japan; [5]The Second Department of Internal Medicine, Tottori University, Yonago, Tottori 683-8503, Japan; [6]Department of Molecular and Cell Genetics, School of Life of Sciences, Faculty of Medicine, Tottori University, Yonago, Tottori 683-8503, Japan; [7]Department of Pulmonary and Mediastinal Pathology, Armed Forces Institute of Pathology, Washington, DC 20306-6000, USA

**Summary** Somatic cells express genes that suppress telomerase activity and these genes may be inactivated in tumour cells. We postulated that cancer cells acquire immortality by activation of telomerase by the loss of such a gene. We have reported recently that a telomerase repressor gene may be located on 10p15.1 by deletion mapping using microcell-mediated chromosome transfer (MMCT), radiated microcell fusion (RMF), fluorescent in situ hybridization (FISH) and STS analysis. To independently confirm this result, we correlated expression of RNA component of telomerase (hTR) as a marker of telomerase expression by in situ hybridization with allelic loss in pulmonary carcinoid tumours. Unlike most malignant tumours, pulmonary carcinoids (which are low-grade malignant tumours) are heterogeneous for telomerase expression. Loss of 5 closely spaced polymorphic markers on 10p15.1, especially D10S1728, were highly correlated with hTR expression. In an additional experiment, 10p15.1 showed higher and more significant correlation than any region of 3p where it has been predicted as another chromosomal location of telomerase repressor with allelic loss of the region. Our findings strongly suggest that 10p15.1 harbours a gene involved in repression of telomerase RNA component in human somatic cells and each putative repressor (on 3p and 10p) may act independently. © 2001 Cancer Research Campaign http://www.bjcancer.com

**Keywords**: hTR; in situ hybridization; mapping; chromosome 10p15; telomerase repressor gene; loss of heterozygosity

Telomerase is an enzyme which adds repeated telomere sequences (TTAGGG) to chromosome ends and has an RNA component complementary to the telomeric repeat sequence (Moyzis et al, 1988; Zakian, 1989). Its catalytic protein component synthesizes telomeric DNA directly onto the ends of chromosomes by the process of reverse transcription of the RNA template (Greider and Blackburn, 1989; Bhattacharyya and Blackburn, 1994; Feng et al, 1995; Kirk et al, 1997). Telomerase activity has been detected in immortalized and tumour cells in vitro and in primary tumour tissues, most fetal tissues, and normal adult male reproductive cells and represents an important difference between normal somatic cells and cancer cells (Kim et al, 1994; Hiyama et al, 1995; Rhyu, 1995; Wright et al, 1996; Shay and Bacchetti, 1997; Sedivy, 1998). Telomerase activity in lysates extracted from tumour cells and tumour tissues is not inhibited by mixture with cell lysates from normal cells, leading to the hypothesis that normal cells contain and express genes which repress the telomerase activity of tumour cells. One approach to map putative telomerase repressor genes to specific chromosomes is the identification of deletions in genome of telomerase-positive cells and tissues. We have previously reported that a senescence-inducing gene on normal human chromosome 3p represses telomerase activity in human renal cell carcinoma cells through down-regulation of the catalytic subunit gene of human telomerase (hEST2/hTERT) (Koi et al, 1989; Shimizu et al, 1990; Ohmura et al, 1995) and, furthermore, the location of the telomerase-repressor gene has been narrowed to the 6 regions in 3p12–3p24.3 (Yashima et al, 1997; Mehle et al, 1998; Tanaka et al, 1998; Vieten et al, 1998; Cuthbert et al, 1999). Of interest, Steenbergen et al revealed that the loss of heterozygosity (LOH) at either chromosome 3p or 10p loci correlated with the onset of telomerase activation during immortalization of human keratinocytes, raising the possibility that a gene on chromosome 10p also may function as a telomerase repressor. Moreover, loss of heterozygosity (LOH) on 10p has been observed in multiple human cancers such as gliomas, oesophageal carcinomas, and melanomas (Robertson et al, 1999), prompting us to focus on this chromosomal arm. We recently presented evidence that a repressor for hTERT was located on 10p15.1 (between markers WI-4752 and D10S1728) (Nishimoto et al, 2001).

We chose hTR expression as a marker of telomerase expression because hTERT shows relatively weak and unstable signals by in situ hybridization. To investigate the relationship between hTR expression and LOH we had to identify tumours which were heterogeneous for telomerase activity. While most tumours are strongly positive for hTR expression, we identified heterogenous

*Received 10 April 2001*
*Revised 10 July 2001*
*Accepted 1 August 2001*

*Correspondence to:* AF Gazdar expression in pulmonary carcinoids which are low-grade neuroendocrine tumours.

### MATERIALS AND METHODS

### A mapping by LOH analysis using STS markers

*Tumour specimens*

A total of 59 neuroendocrine lung tumours were collected from the files of the members of the Pathology Panel of the International Association for the Study of Lung Cancer (IASLC). Histological slides were reviewed by WT, a pathologist familiar with the classification of neuroendocrine lung tumours, and the cases were categorized according to standardized histopathological criteria described previously. The case material consisted of 10 TCs, 11 ACs as low-malignant lung tumours (Onuki et al, 1999).

*Microdissection and DNA extraction*

Serial 5 mm sections were cut from archival, formalin-fixed, paraffin-embedded tissue. All slides were stained with haematoxylin-eosin, and one of the slides was cover slipped. The cover-slipped slide was used as a guide to localize regions of interest for microdissection in the non-cover-slipped slides. Microdissection and DNA extraction were performed as previously described from sections of each sample. Cells from lymph nodes without metastasis or histologically normal lung were used as a source of constitutional DNA from each case. After DNA extraction, 5 µl of the proteinase K digested samples, containing DNA from at least 100 cells, were used for each PCR-based analysis.

*Polymorphic DNA markers and PCR-LOH analysis*

To evaluate LOH and MA in neuroendocrine tumour as low-malignant lesions of SCLC, we used primers flanking 16 di-, tetra-and multinucleotide microsatellite repeat polymorphisms located at the chromosomal locations 10p15 and all the data were compared with those from 3p14.1–21.3. The microsatellite markers and the chromosomal regions analysed were as follows: 10p15 (D10S249, D10S594, D10S602, D10S591, D10S1713, D10S189, D10S1751, D10S1691, AFM154YB4, CHCL.84C03, WI-5574, D10S1779, D10S226, D10S1728, D10S1649, D10S1712, D10S1720, CHCL.117B02, WI-600, D10S547) and 3p14.1–21.3 (D3S1029, D3S1478, KI.CA, D3S1573, ITIH-1, D3S1447, D3S1766). The primer sequences used for LOH studies were obtained from the Genome Database. For all samples, multiplex PCR (up to 8 primers) was performed in the first amplification, followed by uniplex PCR for individual microsatellite markers as previously described. LOH was recognized by visual detection of complete absence of upper or lower allele. MAs were identified by a shift in size of one or both parental alleles compared to constitutional DNA (from normal cells) from the same individual. In the multiplex PCR, 8 markers were amplified in the same reaction using extracted DNA from at least 100 microdissected nuclei. Volumes of 50 µl were used for each multiplex reaction, containing 20 mM TRIS (pH 8.3), 50 mM KCl, 2.5 mM $MgCl_2$, 400 mM of each deoxynucleoside triphosphate (dATP, dCTP, dGTP, dTTP), 0.5 µM of each forward and reverse primer, and 3.5 units of AmpliTaq Gold (Perkin Elmer, Branchburg, NJ). The first PCR product was then diluted 1:10 in double distilled sterile water. The second PCR reaction was performed in 10 µl reaction volume containing 20 mM Tris (pH 8.3), 50 mM KCl, 1.5 mM $MgCl_2$, 100 mM of each deoxynucleoside triphosphate, 0.5 µM of forward and reverse primer, 0.25 unit of AmpliTaq polymerase (Gibco-BRL, Alameda, CA), 0.25 µl of ($\alpha$-$^{32}$P) dCTP (3000 Ci mol$^{-1}$, 10 mCi ml$^{-1}$), (Amersham Life Sciences, Arlington Heights, IL), and 2 µl of diluted first PCR product. In both PCR reactions, a 'touch-down' PCR method was performed. Briefly, after initial denaturation at 94˚C for 4 to 8 min, 11 cycles each consisting of denaturation at 95˚C for 20 s, annealing at 65–56˚C for 55 s and extension at 72˚C for 20 s were performed, followed by an additional 24 cycles which included denaturation at 90˚C for 20 s, annealing at 55˚C for 20 s and extension at 72˚C for 20 s. Then, a 5 µl aliquot of each second PCR reaction was diluted 1:4 with loading buffer, heat denatured, and separated by electrophoresis on a denaturing 6% polyacrylamide gel containing 7 M urea. A water blank control was included in all gels. LOH was scored in informative cases by visual detection of complete absence of the one allele.

### hTR expression in situ hybridization (ISH)

To compare the result of LOH analysis using STS markers with hTR expression, we performed cRNA in situ hybridization in the low-malignant grade of lung tumour (TCs and ACs).

*Preparation and prehybridization of tissue sections for ISH*

Paraffin-embedded (5 mm) tissue sections of 6 tumour-negative and 4 tumour-positive lymph nodes were cut onto Superfrost/Plus slides (Fisher Scientific, Pittsburgh, PA, USA). The sections were deparaffinized, rehydrated in phosphate-buffered saline (PBS), and treated with proteinase K (20 mg ml$^{-1}$) in 50 mM Tris-HCL (pH 7.5), 5 mM EDTA for 20 min at room temperature. After rinsing for 5 min in PBS, sections were postfixed in 4% paraformaldehyde/PBS, rinsed in water, and acetylated in freshly prepared 0.25% acetic anhydride/0.1 M triethanolamine for 10 min. The slides were dehydrated in gradually increasing concentrations of ethanol prior to hybridization.

*Probe preparation for ISH*

The plasmid pGEM-5Zf(+) (Promega Corp., Madison, WI, USA) containing a human telomerase RNA (hTR) complementary DNA (560 nucleotides), obtained from Geron Corp, Menlo Park, CA, USA, was used as a template to generate sense and antisense probe. [$^{35}$S]UTP-labelled single-stranded RNA probes were synthesized according to manufacturer's (Ambion, Inc, Austin, TX, USA) conditions. Transcripts were alkali hydrolysed to generate probes with an average length of 200 nucleotides for efficient hybridization, purified using G-50 column (Boehringer Mannheim Corp, Indianapolis, IN, USA), and precipitated in ethanol. The probes were resuspended in 30 µl of 100 mM dithiothritol. The specific activity of the radiolabelled probes, aliquoted and stored at –80˚C until use, was approximately $3 \times 10^7$ cpm mg$^{-1}$ template DNA.

*Hybridization and washing procedures*

Sections were hybridized overnight at 52˚C in 50% deionized formamide, 0.3 M NaCl, 20 mM TRIS-HCl (pH 7.5), 5 mM EDTA, 10 mM $NaH_2PO4$ (pH 8.0), 10% dextran sulfate, 1 × Denhardt's, 500 mg ml$^{-1}$ total yeast RNA, 10 mM dithiothritol, and 50000 cpm ml$^{-1}$ $^{35}$S-labelled cRNA probe. The tissue was subjected to stringent washing at 50˚C in 5 × SSC, 10 mM dithiothritol for 30 min, at 65˚C in 50% formamide, 2 × SSC, 10 mM DTT for 20 min

© 2001 Cancer Research Campaign and washed twice at 37˚C in 0.4 M NaCl, 10 mM Tris-HCl (pH 7.5), 5 mM EDTA for 10 min before treatment with 20 mg $ml^{-1}$ RNase A at 37˚C for 30 min. Following washes in 2 × SSC and 0.1 × SSC for 10 min at 37˚C, the slides were dehydrated and dipped in Kodak NTB-2 nuclear track emulsion and exposed for 3 wk in light tight boxes with dessicant at 4˚C. The microautradiographs were developed in Kodak Dektol developer (3.5 min), washed in water (20 s), fixed in Kodak fixer (7 min), rinsed in water, counterstained in haematoxylin and eosin. ISH for hTR was performed as described previously (Yashima et al, 1997). To confirm the presence of intact RNA, replicate slides from each sample utilized for hTR expression were also tested for expression of a housekeeping gene, glyceraldehyde 3-phosphate dehydrogenase (GAPDH). The Xba/HindIII fragment from the GAPDH cDNA in pBR322 obtained from the American Type Culture Collection, Rockville, MD, was subcloned into pBluescript. Intense GAPDH hybridization signals were present in all sections studied.

hTR expression was scored as follows: weak – faint expression, detected by brightfield examination at high magnification, but which was detected at low magnification by darkfield examination; moderate – expression readily recognized by brightfield examination at low magnification; high – expression at a level considerably greater than those present in normal. We estimated each level of hTR expression as positive because even weakly positive signals are visually distinguishable, compared with negative ones in ISH.

### RESULTS

### Comparison of hTR expression with LOH analyses

To confirm that the region of interest was between markers D10S226 and D10S547 on 10p15.1, and to narrow this region, we performed LOH analysis by PCR in pulmonary low-malignant tumours including low-grade typical carcinoid (TC) and the more malignant atypical carcinoids (AC) (Travis et al, 1998). Because there were no significant differences between TC and AC, we combined the data. LOH with one or more markers was present in 53% (9/17) of telomerase positive in our samples. Also we compared the data from 10p15 with those from 3p14.2–21.3 because we and others have reported a possibility that a telomerase repressor may be harboured at 3p as well. Represented in Figure 1, in 21 samples of TCs and ACs, 6 LOHs, 2 microsatellite alterations (MAs), 4 not informative cases, and 10 cases without LOH were observed with marker D10S1728, and 2 LOHs were observed with other sequence tagged site (STS) markers on 10p15. All the LOHs and 1 MA concorded with the expression of hTR ISH. hTR expression and LOH in 10p15.1 showed a high concordance (13 out of 15 samples: 87%). On the other hand, 9 out of 17 (53%) in 3p21.2–21.3 and 6 out of 10 (60%) in 3p14.2–21.2 showed a little lower concordance with hTR expression than that of 10p15.1 (Figure 2). Furthermore, comparison between LOH and hTR expression with only a marker D10S1728 revealed 81% of concordance in lung neuroendocrine (NE) tumours. In small-cell lung carcinomas (SCLCs), 15 out of 18 (83.3%) in 10p15.1 (D10S226, D10S1728, D10S1649, D10S1712, and D10S1720) as markers which are located within zero genetic distance) showed LOH and 3 out of 18 showed MAs. In large cell neuroendocrine carcinomas (LCNECs), 14 out of 15 (93.3%) with the same markers of 10p15 showed LOH and 1 out of 15 showed MA. Each level (weak, moderate and high signals) of hTR expression did not significantly correlate with the grades of carcinoid tumours. Additionally, LOH analysis using D10S1728 which showed the highest concordance as the hot spot was performed in 132 lung cancer cell lines but no homozygous deletions were recognized.

### DISCUSSION

Telomerase activity is detected in most human cancer tissues and immortalized cells and is almost universally required for the long-term growth of cancer cells. We and others previously reported that the activation of telomerase is strongly associated with the development of human hepatocellular carcinomas (HCCs) (Miura et al, 1997). Escape from the cellular senescence programme is a

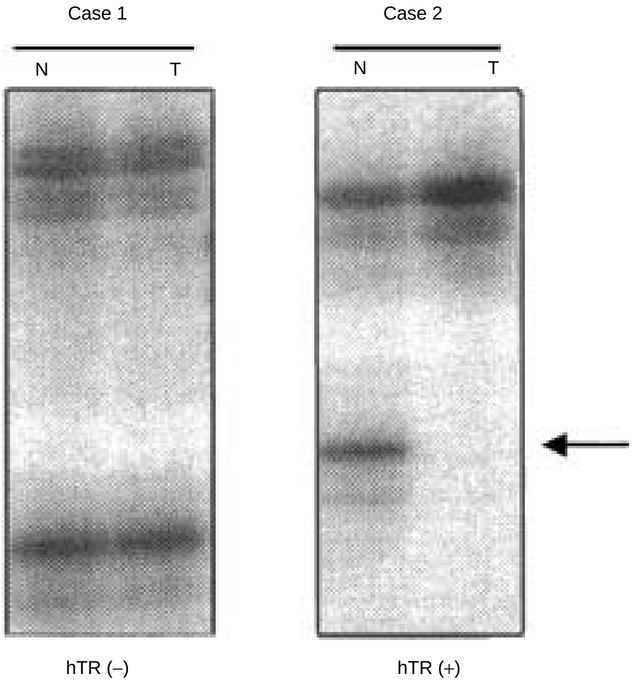

**Figure 1** Microsatellite analyses using D10S1728 in lung carcinoids. Two representatives in 21 cases are illustrated. In case 1, lacking hTR expression, no allelic loss is present. In case 2, positive for hTR expression, the lower allele is absent, indicating alleic loss (LOH). N = normal tissue, T = tumour

3p LOH (*n* = 21)

| 10p15.1 LOH | – | + |
|---|---|---|
| – | 6 | 7 |
| + | 3 | 5 |

Concordance 11/21 (52%)

10p15.1 LOH (*n* = 15)

| hTR | – | + |
|---|---|---|
| – | 7 | 1 |
| + | 1 | 6 |

Concordance 13/15 (87%)

D10S1728 LOH (*n* =16)

| hTR | – | + |
|---|---|---|
| – | 7 | 0 |
| + | 3 | 6 |

Concordance 13/16 (81%)

3p21.2–21.3 LOH (*n* = 17)

| hTR | – | + |
|---|---|---|
| – | 5 | 3 |
| + | 5 | 4 |

Concordance 9/17 (53%)

3p14.2–21.2 LOH (*n* = 10)

| hTR | – | + |
|---|---|---|
| – | 4 | 2 |
| + | 2 | 2 |

Concordance 6/10 (60%)

**Figure 2** Correlation of 10p with 3p for hTR expression by ISH. Allelic losses with 10p15.1 markers in carcinoid lung tumours resulted in higher concordance with hTR expression (87%) than chromosome 3p markers. No significant correlation between 3p and 10p was demonstrated. D10S1728 showed high concordance (81%) with hTR expression than any other markers

 *© 2001 Cancer Research Campaign*

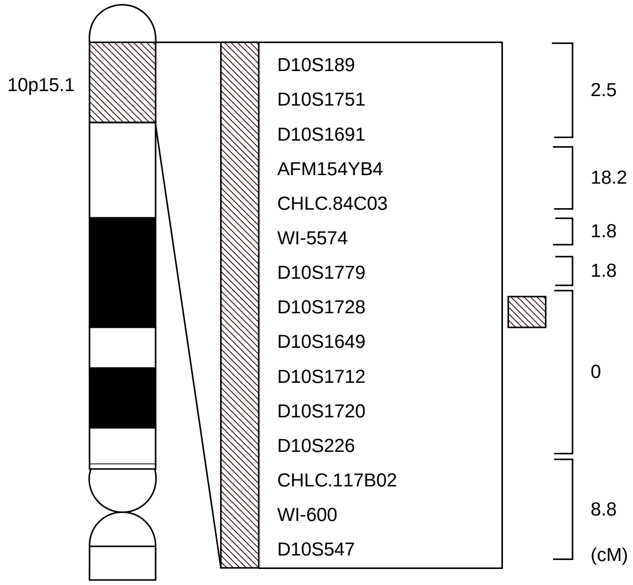

**Figure 3** The summary of this series of mapping. We identified a hot spot (polymorphic marker D10S1728 at 10p15.1) as a functional region whose allelic loss correlates with up-regulation of telomerase activity in lung carcinoids rate-limiting step during carcinogenesis (Norwood et al, 1974; Sager, 1991; Campisi, 1997; Chiu and Harley 1997; Pereira-Smith et al, 1998). We have recently reported the presence of another senescence-inducing gene (Sasaki et al, 1994; Ohmura et al, 1995; Tanaka et al, 1998) that also functions as a telomerase repressor on human chromosome 3p (Holt et al, 1997; Meyerson et al, 1997; Nakamura et al, 1997, 1998). In the case, telomerase repression was due to down-regulation of the gene encoding the catalytic subunit of human telomerase (hEST2/hTERT) (Horikawa et al, 1998). In other cases, telomerase repression during cell differentiation was associated with down-regulation of the RNA component of telomerase (hTR) (Bickenback et al, 1998). Given that LOH at either 3p or 10p was associated with telomerase activation (Steenbergen et al, 1996), the 2 possible telomerase-repressor genes on 3p and 10p may be part of the same cascade of telomerase regulation. Alternatively, as reported in this study, the repressors may have independent, but synergistic functions to regulate the repression pathway of telomerase. Further studies will be needed to determine the molecular basis for telomerase repression by the 10p gene and its functional relationship to the 3p gene. Nonetheless, our results provide new insights into the mechanism of telomerase regulation by demonstrating that the repression of telomerase activity in normal human somatic cells is under multifactorial control, which involves at least 2 telomerase-repressor genes (Oshimura and Barrett, 1997).

Relatively frequent alterations and deletions of chromosome 10p have been identified in many human tumours including glioblastoma multiforme (GBM) (Bigner et al, 1990; Steck et al, 1995; Tsuda et al, 1995; Voesten et al, 1997; Kon et al, 1998), malignant melanoma (MM) (Isshiki et al, 1993), and meningioma (Rempel et al, 1993). Since human glioblastoma hybrid cells containing an entire chromosome 10 or major portions of chromosome 10 exhibited a suppression in their ability to grow anchorage independently and to form tumours in nude mouse (Pershouse et al, 1993), the presence of suppressor gene in chromosome 10p has been suggested. In high-grade astrocytomas such as glioblatoma multiforme (GBM), 3 distinct regions of frequent chromosome 10 deletion have been identified, and one of them is the distal half of the short arm defined by markers D10S33, D10S28, and D10S34 (Karlbom et al, 1993; Steck et al, 1995). This indicates that several distinct tumour suppressor genes may be present on chromosome 10 and may contribute to the progression of malignant tumours. Further investigation has also indicated that LOH on 10p was frequently identified in GBM and MM, and 10p as well as 3p encodes genes involved in telomerase regulation (Steenbergen et al, 1996). 10p15 was structurally altered in malignant melanoma (10pter-p13) and GBM (10pter-p14) and is included in a candidate of locus (10pter-q11) (Sanchez et al, 1996) has pointed out that a telomerase repressor may exist. Frequent LOH on 10p has not been reported in human HCCs. This may suggest that the telomerase-repressor/senescence-inducing gene on 10p is implicated in development of only a fraction of HCCs or fewer STS markers in chromosome 10p than those in other chromosomes have kept the detection of LOH away from us. Actually, we detected 67% of LOH (4/6) at D10S1649 in HCCs (unpublished). Conversely, it is plausible that the 10p15.1 gene is involved in human cancers other than HCC, such as neuroendocrine lung tumour (we detected 56% of LOH (5/9) at D10S1728 and 63% of LOH (7/11) at D10S1712 in lung squamous cell carcinoma). Of interest is whether a putative tumour suppressor gene(s) on 10p for gliomas and oesophageal cancers is identical to the telomerase-repressor/senescence-inducing gene described in this study. As an additional experiment, we performed allelic analyses in microdissected lung tumours in order to confirm whether the hot spot from deletion mapping by MMCT, RMF, and LOH analyses using STS markers can be still identified as a hot spot. Interestingly, as these hot spots showed a higher concordance with the detection of telomerase activity in lung tumours than any part of 3p, though it is true that 3p still shows high concordance (71%) with telomerase activity, a series of our study may indicate that the gene lying around the region of our interest may be able to act as a repressor of hTR and/or transcriptional repressor of hTERT (Nakamura et al, 1997; Ulaner et al, 1998).

### ACKNOWLEDGMENTS

This work was supported by grants from Cancer Research from the Ministry of Education, Science and Culture, Japan; a grant for a Comprehensive 10-Year Strategy for Cancer Control from the Ministry of Health and Welfare of Japan, and by a Specialized Program of Research Excellence (SPORE) P50CA70907 from the National Cancer Institute, Bethesda, MD, USA.

### REFERENCES

Bhattacharyya A and Blackburn EH (1994) Architecture of telomerase RNA. *EMBO J* **13**: 5721–5723

Bickenbach JR, Vormwald DV, Bachor C, Bleuel K, Schnapp G and Boukamp P (1998) Telomerase is not an epidermal stem cell marker and is downregulated by calcium. *J Invest Dermatol* **111**: 1045–1052

Bigner SH, Mark J and Bigner DD (1990) Cytogenetics of human brain tumors. *Cancer Genet Cytogenet* **47**: 141–154

Campisi J (1997) The biology of replicative senescence. *Eur J Cancer* **33**: 703–709

Chiu C-P and Harley CB (1997) Replicative senescence and cell immortality: the role of telomeres and telomerase. *Proc Soc Exp Biol Med* **214**: 99–106

Cuthbert AP, Bond J, Trott DA, Gill S, Broni J, Marriott A, Khoudoli G, Parkinson EK, Cooper CS and Newbold RF (1999) Telomerase repressor sequences on chromosome 3 and induction of permanent growth arrest in human breast cancer cells. *J Natl Cancer Inst* **91**: 37–45

*© 2001 Cancer Research Campaign* 

Feng J, Funk WD, Wang SS, Weinrich SL, Avilion AA, Chiu CP, Adams RR, Chang E, Allsopp RC, Yu J, Le S, West MD, Harley CB, Andrews WH, Greider CW and Villeponteau B (1995) The RNA component of human telomerase. *Science* **269**: 1236–1241

Greider CW and Blackburn EH (1989) A telomeric sequence in the RNA of Tetrahymena telomerase required for telomere repeat synthesis. *Nature* **337**: 331–337

Hiyama K, Hirai Y, Kyoizumi S, Akiyama M, Hiyama E, Piatyszek MA, Shay JW, Ishioka S and Yamakido M (1995) Activation of telomerase in human lymphocytes and hematopoietic progenitor cells. *J Immunol* **155**: 3711–3715

Holt SE, Wright WE and Shay JW (1997) Multiple pathways for the regulation of telomerase activity. *Eur J Cancer* **33**: 761–766

Horikawa I, Oshimura M and Barrett JC (1998) Repression of the telomerase catalytic subunit by a gene on human chromosome 3 that induces cellular senescence. *Mol Carcinog* **22**: 65–72

Isshiki K, Elder DE, Guerry D and Linnenbach AJ (1993) Chromosome 10 allelic loss in malignant melanoma. *Genes Chromosomes Cancer* **8**: 178–184

Karlbom AE, James CD, Boethius J, Cavenee WK, Collins VP, Nordenskjold M and Larsson C (1993) Loss of heterozygosity in malignant gliomas involves at least three distinct regions on chromosome 10. *Hum Genet* **92**: 169–174

Kim NM, Piatyszek MA, Prowse KR, Harley CB, West MD, Ho PLC, Coviello GM, Wright WE, Weinrich SL and Shay JW (1994) Specific association of human telomerase activity with immortal cells and cancer. *Science* **226**: 2011–2015

Kirk KE, Harmon BP, Reichardt IK, Sedat JW and Blackburn EH (1997) Block in anaphase chromosome separation caused by a telomerase template mutation. *Science* **275**: 1478–1481

Koi M, Shimizu M, Morita H, Yamada H, Satoh H, Barrett JC and Oshimura M (1989) Normal human chromosome 11 suppresses tumorigenicity of human cervical tumor cell line SiHa. *Mol Carcinogen* **2**: 12–21

Kon H, Sonoda Y, Kumabe T, Yoshimoto T, Sekiya T and Murakami Y (1998) Structural and functional evidence for the presence of tumour suppressor genes on the short arm of chromosome 10 in human gliomas. *Oncogene* **16**: 257–263

Mehle C, Lindblom A, Ljungberg B, Stenling R and Roos G (1998) Loss of heterozygosity at chromosome 3p correlates with telomerase activity in renal cell carcinoma. *Int J Oncol* **13**: 289–295

Meyerson M, Counter CM, Eaton EN, Ellisen LW, Steiner P, Caddle SD, Ziaugra L, Beijersbergen RL, Davidoff MJ, Liu Q, Bacchetti S, Haber DA and Weinberg RA (1997) hEST2, the putative human telomerase catalytic subunit gene, is up-regulated in tumor cells and during immortalization. *Cell* **90**: 785–795

Miura N, Horikawa I, Nishimoto A, Ohmura H, Ito H, Hirohashi S, Shay JW and Oshimura M (1997) Progressive telomere shortening and telomerase reactivation during hepatocellular carcinogenesis. *Cancer Genet Cytogenet* **93**: 56–62

Moyzis RK, Buckingham JM, Cram LS, Dani M, Daven LL, Jones MD, Meyne J, Ratliff RL and Wu JR (1988) A highly conserved repetitive DNA sequence, (TTAGGG)n, present at the telomeras of human chromosomes. *Proc Natl Acad Sci USA* **85**: 6622–6626

Nakamura TM, Morin GB, Chapman KB, Weinrich SL, Andrews WH, Lingner J, Hughes TR, Shevchenko A, Mann M, Lundblad V and Cech TR (1997) Telomerase catalytic subunit homologs from fission yeast and human. *Science* **276**: 561–567

Nakayama J-I, Tahara H and Tahara E (1998) Telomerase activation by hTRT in human normal fibroblasts and hepatocellular carcinomas. *Nat Genet* **18**: 65–68

Nishimoto A, Miura N, Horikawa I, Kugoh H, Murakami Y, Hirohashi S, Kawasaki H, Gazdar AF, Shay JW, Barrett JC and Oshimura M (2001) Functional evidence for a telomerase repressor gene on human chromosome 10p15.1. *Oncogene* **20**: 828–835

Norwood TH, Pendergrass WR, Sprague CA and Martin GM (1974) Dominance of the senescent phenotype in heterokaryons between replicative and post-replicative human fibroblast-like cells. *Proc Natl Acad Sci USA* **71**: 2231–2235

Ohmura H, Tahara H, Suzuki M, Ide T, Shimizu M, Yoshida MA, Tahara E, Shay JW, Barrett JC and Oshimura M (1995) Restoration of the cellular senescence program and repression of telomerase by human chromosome 3. *Jpn J Cancer Res* **86**: 899–904

Onuki N, Wistuba II, Travis WD, Virmani AK, Yashima K, Brambilla E, Hasleton P and Gazdar AF (1999) Genetic changes in the spectrum of neuroendocrine lung tumors. *Cancer* **85**: 600–607

Oshimura M and Barrett JC (1997) Multiple pathways to cellular senescence: role of telomerase repressors. *Eur J Cancer* **33**: 710–715

Pereira-Smith OM and Smith JR (1988) Genetic analysis of indefinite division in human cells: identification of four complementation groups. *Proc Natl Acad Sci USA* **85**: 6042–6046

Pershouse MA, Stubblefield E, Hadi A, Killary AM, Yung WK and Steck PA (1993) Analysis of the functional role of chromosome 10 loss in human glioblastomas. *Cancer Res* **55**: 5043–5050

Rempel SA, Schwechheimer K, Davis RL, Cavenee WK and Rosenblum (1993) Loss of heterozygosity for loci on chromosome 10 is associated with morphologically malignant meningioma progression. *Cancer Res* **53**: 2386–2392

Rhyu MS (1995) Telomeres, telomerase, and immortality. *J Natl Cancer Inst* **87**: 884–894

Robertson GP, Herbst RA, Nagane M, Huang HS and Cavenee WK (1999) The Chomosome 10 monosomy common in human melanomas results from the loss of two separate tumor suppressor loci. *Cancer Res* **59**: 3596–3601

Sager R (1991) Senescence as a mode of tumor suppression. *Environ Health Perspect* **93**: 59–62

Sanchez Y, Lovell M, Marin MC, Wong PE, Wolf-Ledbetter ME, McDonnell TJ and Killary AM (1996) Tumor suppression and apoptosis of human prostate carcinoma mediated by a genetic locus within human chromosome 10pter-q11. *Proc Natl Acad Sci USA* **93**: 2551–2556

Sasaki M, Honda T, Yamada H, Wake N, Barrett JC and Oshimura M (1994) Evidence for multiple pathways to cellular senescence. *Cancer Res* **54**: 6090–6093

Sedivy JM (1998) Can ends justify the means?: telomeres and the mechanisms of replicative senescence and immortalization in mammalian cells. *Proc Natl Acad Sci USA* **95**: 9078–9081

Shay JW and Bacchetti S (1997) A survey of telomerase activity in human cancer. *Eur J Cancer* **33**: 787–791

Shimizu M, Yokota J, Mori N, Shuin T, Shinoda M, Terada M and Oshimura M (1990) Introduction of normal chromosome 3p modulates the tumorigenicity of a human renal cell carcinoma cell line YCR. *Oncogene* **5**: 185–194

Steck PA, Ligon AH, Cheong P, Alfred-Yung WK and Pershouse MA (1995) Two tumor suppressive loci on chromosome 10 involved in human glioblastomas. *Genes Chromosomes Cancer* **12**: 255–261

Steenbergen RDM, Walboomers JMM, Meijer CJLM, van der Raaij-Helmer EM, Parker JN, chow LT, Broker TR and Snijders PJF (1996) Transition of human papillomavirus type 16 and 18 transfected human foreskin keratinocytes towards immortality: activation of telomerase and allele losses at 3p, 10p, 11q and/or 18q. *Oncogene* **13**: 1249–1257

Tanaka H, Shimizu M, Horikawa I, Kugoh H, Yokota J, Barrett JC and Oshimura M (1998) Evidence for a putative telomerase repressor gene in the 3p14.2-p21.1 region. *Genes Chromosomes Cancer* **23**: 123–133

Travis WD, Rush W, Flieder DB, Falk R, Fleming MV, Gal AA and Koss MN (1998) Survival analysis of 200 pulmonary neuroendocrine tumors with clarification of criteria for atypical carcinoid and its separation from typical carcinoid *Am J Surg Pathol* **22**: 934–944

Tsuda T, Marinetti MR, Masuelli LI and Cutler ML (1995) The Ras suppressor RSU-1 localizes to 10p13 and its expression in the U251 glioblastoma cell line correlates with a decrease in growth rate and tumorigenic potential. *Oncogene* **11**: 397–403

Ulaner GA, Hu JF, Vu TH, Giudice LC and Hoffman AR (1998) Telomerase activity in human development is regulated by human telomerase reverse transcriptase (hTERT) transcription and by alternate splicing of hTERT transcripts. *Cancer Res* **58**: 4168–4172

Vieten L, Belair CD, Savelieva L, Julicher K, Brocker F, Bardenheuer W, Schutte J, Opalka B and Reznikoff CA (1998) Minimal deletion of 3p13→14.2 associated with immortalization of human uroepithelial cells. *Genes Chromosomes Cancer* **21**: 39–48

Voesten AM, Bijleveld EH, Westerveld A and Hulsebos TJ (1997) Fine mapping of a region of common deletion on chromosome arm 10p in human glioma. *Gene Chromosome Cancer* **20**: 167–172

Wright WE, Piatyszek MA, Rainey WE, Byrd W and Shay JW (1996) Telomerase activity in human germline and embryonic tissues and cells. *Dev Genet* **18**: 173–179

Yashima K, Piatyszek MA, Saboorian HM, Virmani AK, Brown D, Shay JW and Gazdar AF (1997) Telomerase activity and in situ telomerase RNA expression in malignant and non-malignant lymph nodes. *J Clin Pathol* **50**: 110–117

Zakian VA (1989) Structure and function of telomeres. *Annu Rev Genet* **23**: 579–604

 *© 2001 Cancer Research Campaign*
